# Evolutionary Patterns of Collagen Fiber Arrangement and Calcification in Atherosclerosis

**DOI:** 10.34133/research.0798

**Published:** 2025-07-31

**Authors:** Chunyang Ma, Zhenzhen Jia, Shuaiyin Liu, Xiangyi Liao, Hongyan Kang, Xufeng Niu, Yubo Fan

**Affiliations:** ^1^ Key Laboratory of Biomechanics and Mechanobiology ( Beihang University), Ministry of Education; Key Laboratory of Innovation and Transformation of Advanced Medical Devices, Ministry of Industry and Information Technology; National Medical Innovation Platform for Industry-Education Integration in Advanced Medical Devices (Interdiscipline of Medicine and Engineering); School of Biological Science and Medical Engineering, Beihang University, Beijing 100083, China.; ^2^ School of Engineering Medicine, Beihang University, Beijing 100083, China.

## Abstract

Collagen is a major structural component of human tissues, and its spatial arrangement is critical for maintaining tissue mechanical integrity and regulating cell behavior. In atherosclerosis (AS), collagen fiber arrangement has been implicated in plaque stability and the regulation of vascular calcification, yet its evolution during disease progression and association with other pathological processes remain poorly understood. In this study, male ApoE^−/−^ mice were fed a high-fat diet to establish a model of AS. Histological staining was performed every 5 weeks to analyze the collagen fiber alignment in the aortic arch of mice, along with calcification-related cells, proteins, and growth factors. The experimental results indicate that collagen fiber arrangement underwent gradual disruption and randomization as the disease progressed. Randomized collagen fibers were found to colocalize with inflammatory infiltration, smooth muscle cell phenotypic switching, osteogenic gene expression, and calcification. Therefore, changes in collagen fiber arrangement can be used to determine the presence of AS lesions, delineate their location, monitor disease progression, and assess plaque stability, thereby providing a solid theoretical foundation for the diagnosis and treatment of AS.

## Introduction

Collagen (Col) is the most abundant protein in human body, widely distributed across tissues such as the skin, bones, tendons, and blood vessels [1–5]. Col typically exists in a fibrous form, and its intricate structural arrangement is fundamental to the functional integrity of various organs. For instance, in the skin, Col fibers are arranged in a reticular pattern, providing elasticity and tensile strength [6,7]. In tendons, Col fibers are arranged in parallel, enabling them to withstand higher tensile forces [8–10]. Studies have shown that collagen fiber arrangement (CFA) often undergoes alterations with disease onset or aging, leading to a decline or even loss of organ function. For example, in cancerous regions, Col fibers undergo changes in diameter, cross-sectional shape, and alignment, which modulate behaviors such as cancer cell migration [11–17]. As individuals age, Col fibers in the skin become sparse and loosened, resulting in decreased skin elasticity [18–22]. It is evident that CFA plays a crucial role in disease development and warrants considerable attention.

Atherosclerosis (AS) is a widespread chronic disease, characterized by its lifelong presence and difficulty in curing. Plaque stability is the central issue throughout the progression of AS, drawing considerable interest in the field [23–29]. Current studies have shown that plaque stability is closely related to the concentration and structure of Col in the fibrous cap, as well as to distribution of calcification (Cal) [30–34]. Plaques with low Col concentration and poor orientation, along with diffuse Cal, are highly prone to rupture or detachment, leading to vascular occlusion, myocardial infarction, and other severe cardiovascular events, thus posing a serious risk to human life and health [35,36]. Moreover, Col, serving as a template for Cal, plays a critical regulatory role in its formation, making Col more pivotal in AS [37–39].

Given the important impact of CFA in the development of other diseases and the role of Col in plaque Cal, it is also crucial to conduct in-depth research on CFA in AS [40–43]. However, existing research primarily focuses on the stability of plaques in late stage, with insufficient attention given to the early development of plaques. Plaques gradually develop through a prolonged evolutionary process [44–47]. Investigating the patterns of CFA changes in the early stages of plaque development and their relationship with disease progression can provide a strong theoretical foundation for early diagnosis, timely prevention, and treatment of the disease. Meanwhile, changes in CFA also contribute to a better understanding of alterations in vascular mechanical properties, providing new insights for the treatment of vascular sclerosis.

Therefore, this study systematically analyzed the abnormal changes in CFA using a high-fat diet-fed ApoE^−/−^ mouse model, as well as the factors associated with these changes, providing new scientific evidence and research insights for the diagnosis and treatment of AS, as well as the prevention of severe cardiovascular events.

## Results

### CFA exhibits a colocalization relationship with multiple pathological phenomena

**Fig. 1.**
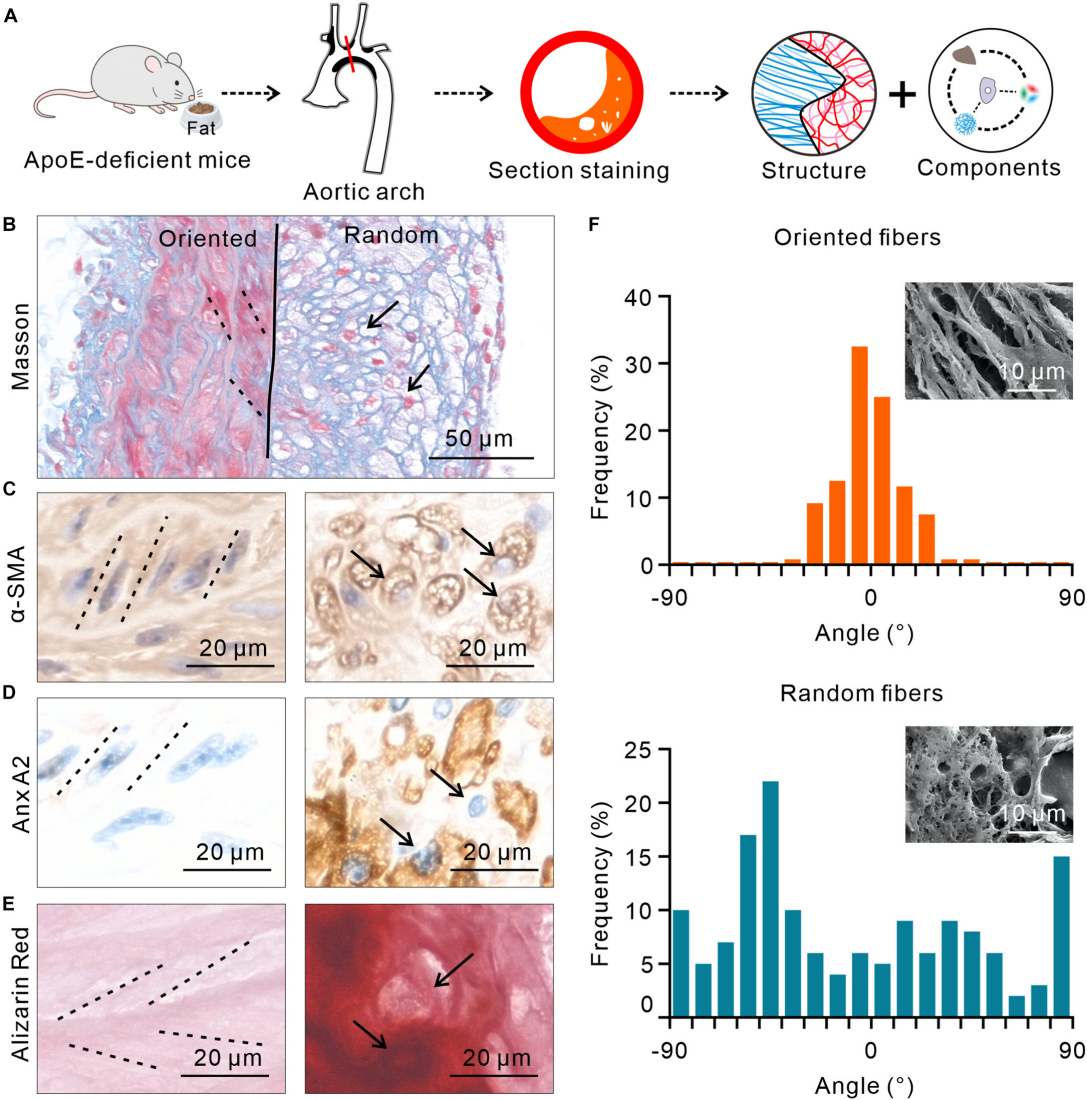
Experimental procedure and the colocalization relationship between CFA and other pathological phenomena. (A) Schematic diagram of the experimental procedure, including steps such as high-fat diet feeding for modeling, harvesting the aortic arch, sectioning and staining, as well as observation and statistical analysis. (B) Masson staining image of the site of AS occurrence. The Col fibers and muscle fibers in healthy area show clear orientation, whereas both are random in diseased area, with a very distinct boundary between the 2 regions. (C) α-SMA IHC of oriented and random regions. The oriented regions show clear directional alignment of SMCs and their nuclei, while the random regions show nearly round-shaped SMCs and nuclei. (D) Anx A2 IHC of the oriented and random regions. The content of Anx A2 is markedly elevated in random regions. (E) Alizarin Red staining image of the oriented and random regions. Cal only occurs in random regions. (F) SEM images and statistical analysis of the fiber angle distribution. CFA differs markedly between the 2 regions (*n* = 100). The staining, IHC, and SEM section images are from 5 different mice, and the angle analysis statistics are derived from the SEM images.

To investigate the patterns of CFA changes in AS and their relationship with other pathological phenomena, a preliminary observation was conducted on areas with plaques (Fig. 1A). Masson staining results show that CFA in healthy regions is oriented (Fig. 1B, black dashed line), while CFA in the diseased area is random (Fig. 1B, black arrow), with a distinct boundary between the 2 regions (Fig. 1B, black solid line). The orientation of muscle fibers is consistent with that of CFA. In oriented regions of CFA, the muscle fibers are also oriented, and their direction is the same. In random regions of CFA, muscle fibers contract into circular shapes and lack orientation. Immunohistochemistry (IHC) for α-SMA (smooth muscle actin) shows that in the oriented regions, smooth muscle cells (SMCs) and their nuclei are oriented (Fig. 1C, black dashed line), while in the random regions, SMCs and their nuclei adopt a circular shape (Fig. 1C, black arrow). IHC for annexin (Anx) A2 shows that there is almost no Anx A2 in oriented regions, while a large amount of Anx A2 is present in random regions (Fig. 1D). Alizarin Red staining shows that Cal occurs in random regions, but not in oriented regions (Fig. 1E). Scanning electron microscope (SEM) images and statistical analysis of fiber orientation reveal that in the aligned region, 98% of the fibers deviate less than 30° from the principal direction, whereas in the random region, only 36% fall within this range (Fig. 1F). In addition, macrophage and metalloproteinase 1 (MMP1) levels were markedly elevated in the random regions compared to the oriented regions (Fig. S1A and B). SMCs lost their alignment in regions with high levels of macrophages and MMP1 (Fig. S1C and D). The abundant presence of MMPs may be one of the reasons that lead to more random CFA. It is evident that the random CFA exhibits a marked colocalization with random SMCs, Anx, Cal, and inflammation.

### The randomization of CFA is an important marker of AS

**Fig. 2.**
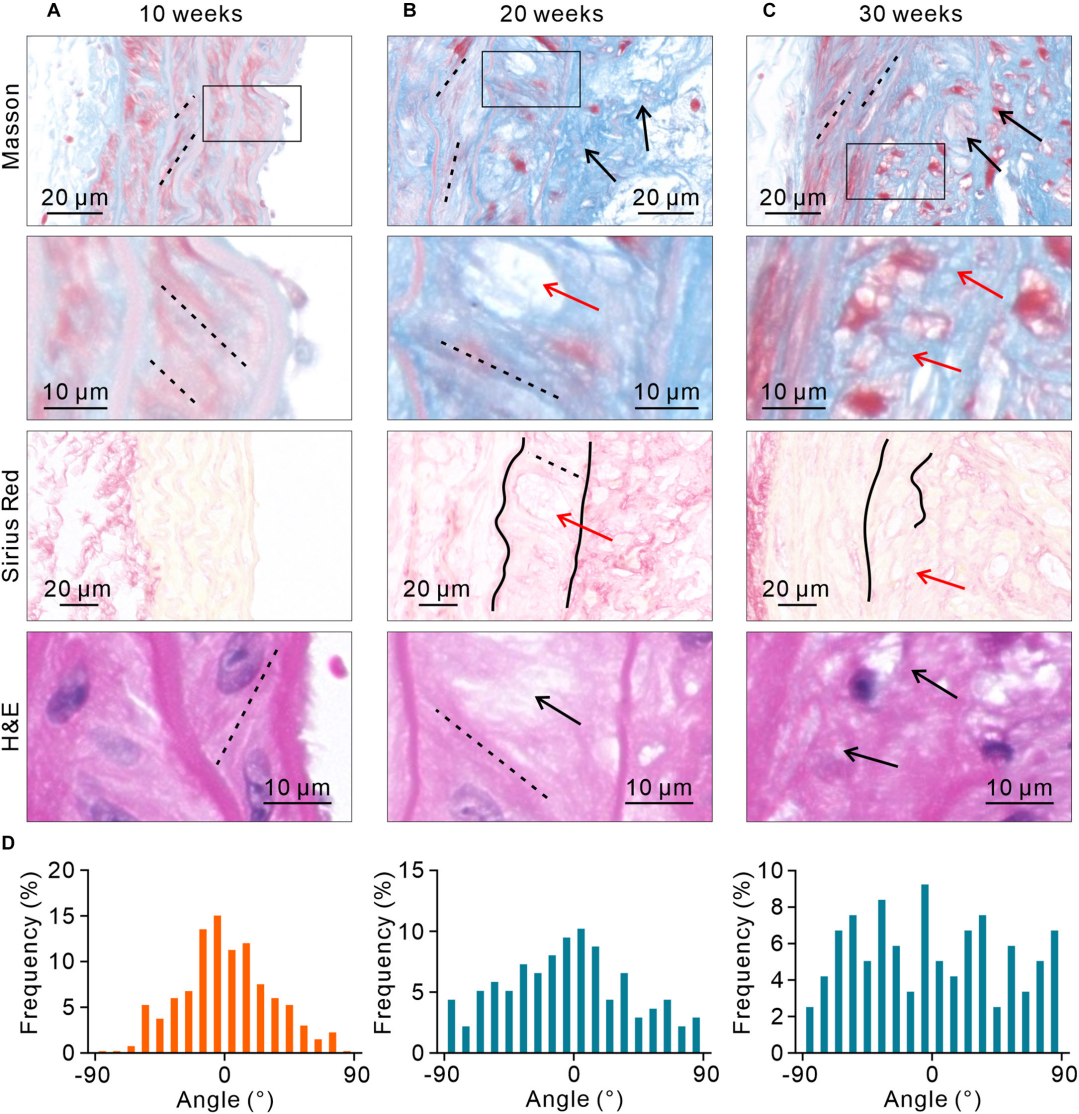
The changes in CFA with the occurrence and development of AS. (A) Masson, Sirius Red, and H&E staining images of the vascular wall at week 10. (B) Masson, Sirius Red, and H&E staining images of the vascular wall at week 20. (C) Masson, Sirius Red, and H&E staining images of the vascular wall at week 30. (D) Statistical analysis of the Col fiber orientation distribution in the innermost layer of media at the 3 stages, showing that CFA in the innermost layer of media gradually becomes random from being oriented (*n* = 100). The staining images at each time point are from the same mouse. A total of 3 mice were used in this experiment, and the angle analysis statistics are derived from the H&E images.

Through Masson’s trichrome, Sirius Red, and hematoxylin and eosin (H&E) staining, a more in-depth observation of the changes in CFA revealed that the randomization of CFA is closely associated with the occurrence and development of AS. By week 10, AS has not yet occurred, and the CFA in each layer of media is oriented (Fig. 2A, black dashed line). By week 20, plaque has appeared, and the CFA within it is random (Fig. 2B, black arrow). In the innermost layer of media, CFA begins to become random (Fig. 2B, red arrow), but some orientation is still preserved (Fig. 2B, black dashed line). In contrast, CFA in the other layers of media still retains its original orientation (Fig. 2B, black dashed line). By week 30, in the innermost layer of media, CFA has become completely random (Fig. 2C, red arrow). The other layers of the media still maintain their orientation (Fig. 2C, black dashed line). The orientation of SMCs is always consistent with that of CFA (Fig. 2A to C). Statistical analysis showed that at week 10, 66% of the fibers were oriented within 30° of the principal direction, compared to 47% at week 20 and 34% at week 30, indicating a progressive loss of fiber alignment over time (Fig. 2D). It is worth noting that lesions in the innermost layer of media only occur in areas with plaques (Fig. S2). In addition, the thickness of the innermost layer of the media also undergoes marked changes, which occur almost synchronously with the changes in CFA (Fig. S3).

### The changes in CFA are related to the phenotypic transformation of SMCs

**Fig. 3.**
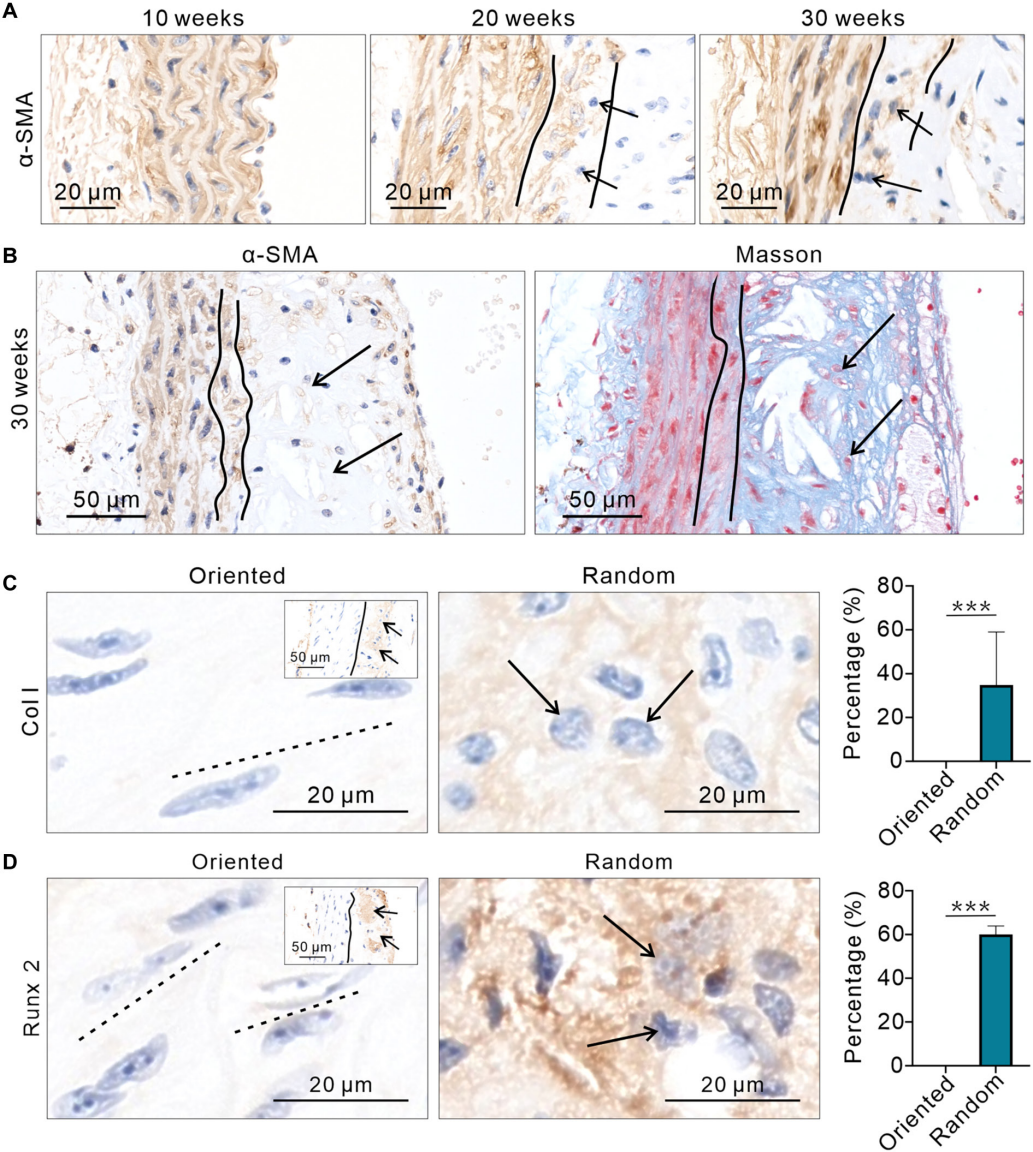
The phenotypic transformation of SMCs. (A) IHC images of α-SMA from 10 to 30 weeks. As the disease progresses, the content of α-SMA in diseased area gradually decreases. (B) Comparison between the IHC images of α-SMA and Masson images. There are still a large number of muscle fibers present in the areas where the content of α-SMA is low. (C) IHC images show that the content of Col I is higher in random area (*n* = 5). (D) IHC images show that the content of Runx 2 is higher in random area (*n* = 5). The same mouse was used for each time point and IHC, with a total of 5 mice used. The significance of differences between groups was analyzed using the *t* test, with ***P < 0.001.

From Masson staining, it can be seen that muscle fibers, like CFA, exhibit a more random trend as the disease progresses. Therefore, IHC of α-SMA was used to conduct a more in-depth analysis of the changes in SMCs. SMCs originally only existed in the media of blood vessels, and after the appearance of plaques, a portion of SMCs entered the plaques. Soon, the expression level of α-SMA in SMCs in plaque began to decrease, indicating that SMCs may have undergone phenotypic transformation (Fig. 3A, right of 2 black lines). The α-SMA in the innermost layer of media showed a gradually decreasing trend, indicating that the phenotype of SMCs in this area is slowly transforming (Fig. 3A, between 2 black lines). From the morphology of the cell nucleus, it can also be seen that directionality is gradually losing (Fig. 3A, black arrow). IHC of α-SMA and Masson staining jointly confirmed the presence of SMCs within the plaque, but the expression level of α-SMA decreased (Fig. 3B, black arrow). Meanwhile, the SMCs in random regions began to express osteogenic-related genes, such as Col I and Runx 2 (Fig. 3C and D). In vitro culture and osteogenic induction of SMCs showed that phenotypic changes lead to morphological alterations in both SMCs and their surrounding matrix, which closely resemble the pathological changes observed in vivo (Fig. S4).

### Cal occurs only in the random regions of CFA

**Fig. 4.**
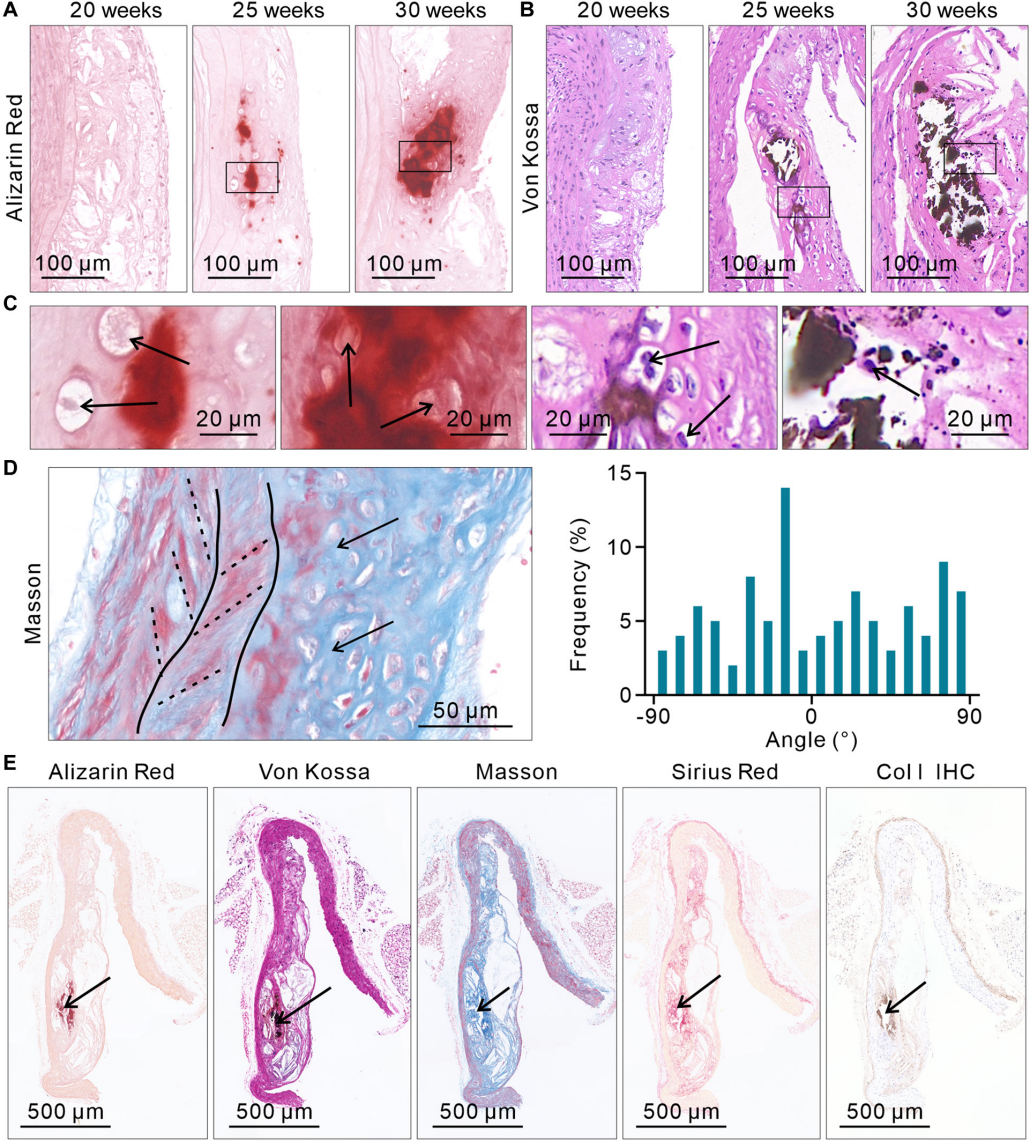
The relationship between Cal and CFA. (A) Development of Cal shown by Alizarin Red staining. Cal first appears at week 25 and rapidly enlarges over the next 5 weeks. (B) Development of Cal shown by Von Kossa staining, with results consistent with those observed in Alizarin Red staining. (C) Magnified images of the Cal site. The matrix and nuclei at the site of Cal are random. (D) Masson staining and statistical analysis of Col fiber orientation distribution, showing that CFA at the site of Cal is random (*n* = 100). (E) Alizarin Red, Von Kossa, Masson, Sirius Red, and Col I IHC images of the vascular wall at week 30, showing marked colocalization between Cal and Col I. The same mouse was used for each time point and IHC, with a total of 5 mice used.

Alizarin Red staining images show that Cal appears at week 25 and becomes quite extensive by week 30 (Fig. 4A). The statistical results of Cal size also confirm that at week 25, the average Cal diameter was 45.05 μm with a mean area of 1.36 × 10^3^ μm^2^. By week 30, the diameter had increased to 163.20 μm, with an area of 12.00 × 10^3^ μm^2^ (Fig. S5). Von Kossa staining shows the same results (Fig. 4B). In the magnified images, it is clearly observed that the matrix at the site of Cal is random, and the nuclei near the Cal site are also round (Fig. 4C, black arrow). Masson staining and statistical analysis of Col fiber orientation distribution show that the CFA at the site of Cal is random (Fig. 4D). No Cal occurs in the oriented regions (Fig. 4D, black dashed line). The Sirius Red, Von Kossa, Masson, Sirius Red, and Col I IHC images show marked colocalization between Cal and Col I (Fig. 4E).

### The content of Anx is higher in CFA randomized regions

**Fig. 5.**
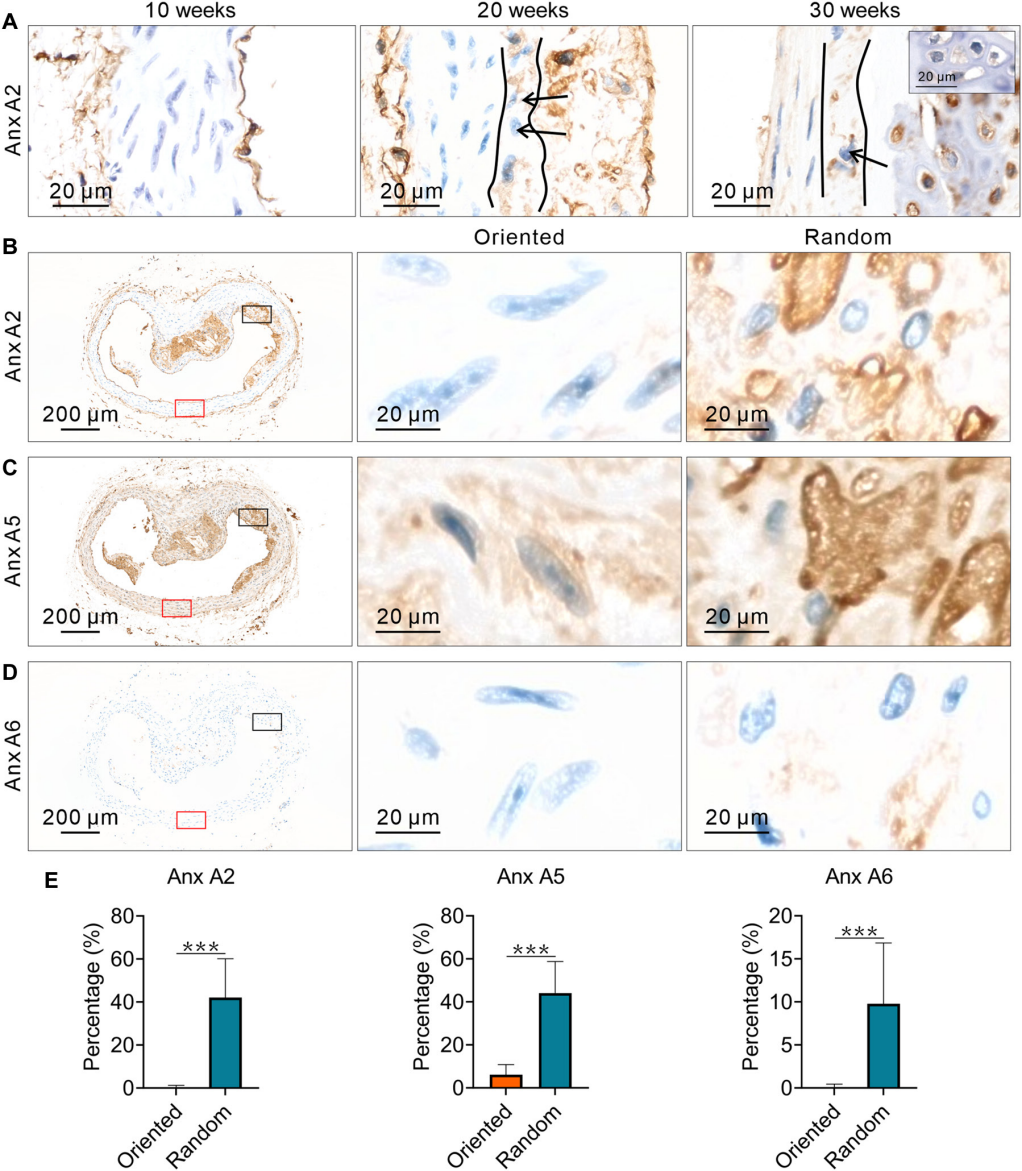
The relationship between Anx and CFA. (A) IHC images of Anx A2 from weeks 10 to 30. Before week 25, the levels of Anx A2 in random regions are markedly elevated, but this phenomenon becomes less pronounced after 25 weeks. (B) IHC images of Anx A2 in oriented and random regions. The oriented regions contain almost no Anx A2, whereas the random regions are densely populated with Anx A2. (C) IHC images of Anx A5 in oriented and random regions. The content of Anx A5 is markedly elevated in random regions. (D) IHC images of Anx A6 in oriented and random regions. The content of Anx A6 is low in both regions, but still slightly higher in random regions. (E) Statistical analysis of the levels of the 3 Anx in oriented and random regions. The content of all 3 Anx is markedly elevated in random regions (*n* = 5). Images for each IHC and each time point are from a single mouse, with a total of 6 mice used. The significance of differences between groups was analyzed using the *t* test, with ***P < 0.001.

Anx is a protein closely associated with Cal on matrix vesicles. Before week 25, the concentration of Anx A2 in random regions is notably higher, but this phenomenon becomes less pronounced after week 25 (Fig. 5A). The decrease in Anx levels during the late stage of AS progression may be due to the reduced number of macrophages. IHC images of CD68 show a marked decrease in macrophage content within the plaques at week 30 (Fig. 5A, inset). Similar patterns were observed for Anx A5 and A6 (Fig. S6). So, the 20th week was given special attention. The results of IHC showed that the content of Anx A2 was markedly elevated in random area (Fig. 5B). The content of Anx A5 in random area was also higher (Fig. 5C). The content of Anx A6 was low in both areas, but it was higher in random area (Fig. 5D). The statistical results show that the levels of the 3 Anx are markedly elevated in random regions (Fig. 5E).

## Discussion

Analyzing pathological issues from the perspective of materials science can offer a novel viewpoint for a deeper understanding of disease progression and provide valuable references for the development of therapeutic approaches. CFA has demonstrated a strong correlation with disease progression and has been utilized in the diagnosis and treatment of various conditions. However, its significance in AS has largely been overlooked. ApoE^−/−^ mice are widely recognized as a standard model for studying AS and are well-suited for investigating CFA changes due to their consistent plaque development and key similarities to human disease. Through modeling and detailed observation, this study reveals that the randomization of CFA serves as a critical marker for the onset and progression of AS, especially closely related to Cal.

Preliminary observations have revealed that in the CFA random area, SMCs and their nuclei are nearly round in shape, with higher levels of macrophages, MMP1, and Anx, as well as the presence of Cal. In contrast, the CFA oriented region does not exhibit these pathological changes. These findings indicate that pathological changes occur exclusively in regions with random CFA, with a clear distinction between these regions and others. This further confirms the crucial role of CFA in the development of AS.

Plaques, as a typical hallmark of pathology, exhibit CFA that remains random throughout their development, in stark contrast to the oriented CFA in healthy regions. Col fibers and muscle fibers in each layer of the media are originally oriented, but following the formation of plaques, the orientation of Col and muscle fibers in the innermost layer of the media begins to decline, ultimately becoming completely random. All these suggest that the randomization of CFA is an important manifestation of the development of AS. The orientation of Col fibers and SMCs remains consistently aligned. IHC images of α-SMA further demonstrate this phenomenon. In addition, both in vivo and in vitro experiments have proved that the rounded SMCs have undergone phenotypic transformation, which may be one of the reasons for the randomization of CFA.

Notably, SMCs exhibit an elongated morphology and a tendency for directional alignment even under standard culture conditions without any external induction, indicating that the orientation of Col fibers is not a prerequisite for SMC alignment. From the perspective of alignment alone, it appears that SMCs exert a greater influence on the organization of Col fibers. However, Col is also essential for SMC adhesion to the culture substrate. Under specific conditions, such as in the presence of MMPs, Col degradation impairs cell attachment, leading SMCs to adopt a nearly rounded shape. These observations suggest a mutually dependent relationship between Col and SMC orientation, which underlies their close correlation.

The sites of Cal are predominantly located at the base of plaques, where both CFA and cells are random. This finding is further confirmed by Masson staining. Although not all CFA random regions show Cal, Cal only occurs in the CFA random areas. This still establishes a close relationship between Cal and random CFA. Further studies have shown a marked colocalization between Cal and Col I, suggesting that these pathological phenomena are closely related to the phenotypic transformation of SMCs.

Anx A2, A5, and A6 are essential proteins present on matrix vesicles, playing a critical role in regulating the influx and efflux of calcium ions, as well as influencing interactions with Col. The levels of these 3 Anx show a strong correlation with CFA. Notably, before week 25, Anx concentrations in random regions are markedly elevated. Although the levels of Anx decrease starting from week 25, the phenomenon becomes less pronounced, yet it remains a valuable indicator. From the IHC staining of CD68, the decrease in Anx levels is closely associated with the reduction in the number of macrophages. Furthermore, as the disease progresses, the content of Anx in the innermost layer of media shows a distinct increasing trend. Therefore, Anx is closely associated with CFA, further highlighting the potential role of CFA in the progression of AS.

Observations also revealed that AS lesions are localized, suggesting that local treatments such as scaffold implantation are viable options. Given that changes in CFA are influenced by MMPs, phenotypic transformation of SMCs, and inflammatory responses, scaffold design could incorporate drugs that inhibit the expression of related genes or suppress inflammatory reactions. The scaffold structure could also be designed to be directional. Additionally, changes in Col type and content are closely related to the occurrence of Cal, indicating that regulating Col could potentially inhibit Cal.

**Fig. 6.**
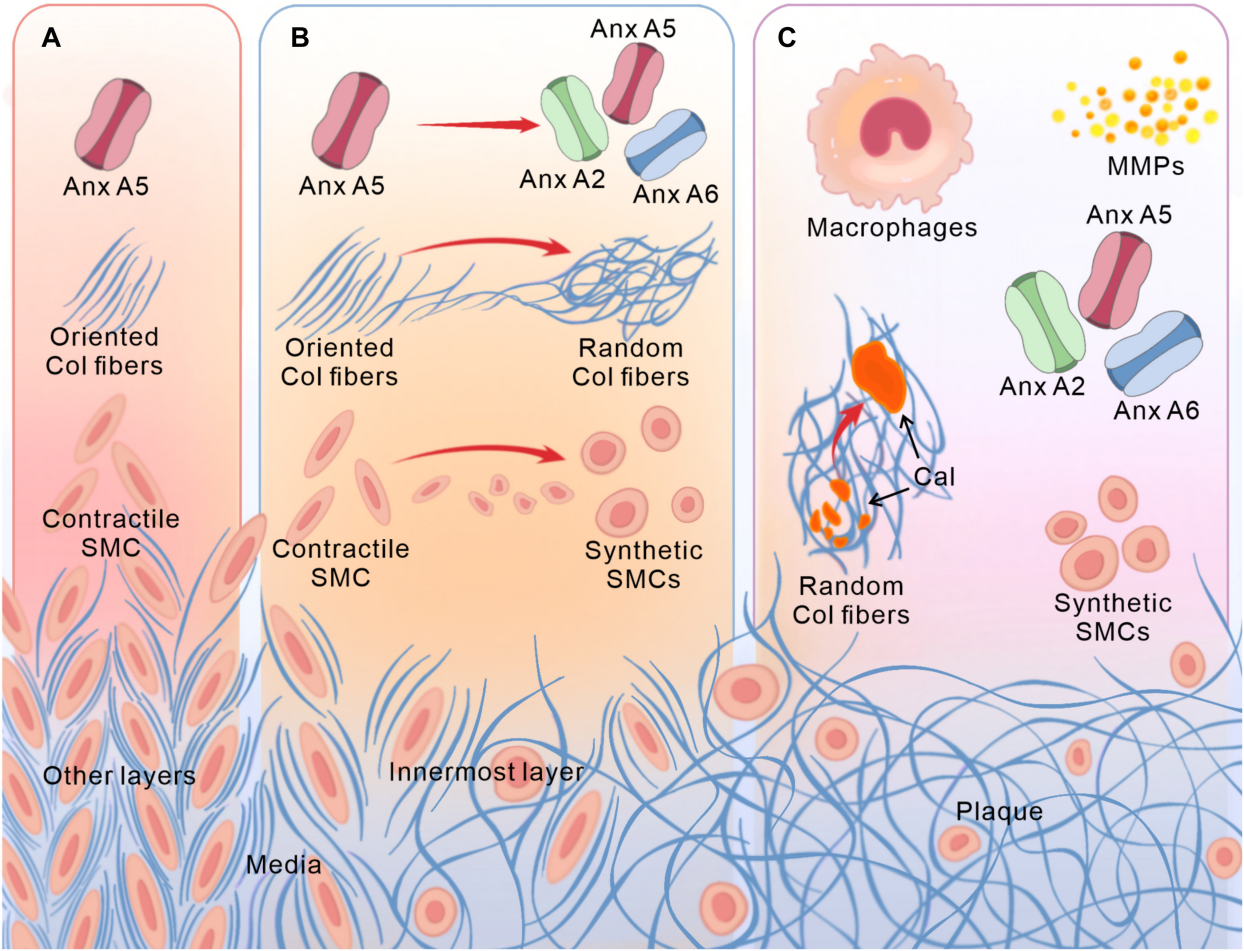
Schematic diagram showing the relationship between CFA and the pathological Cal process. (A) As the disease progresses, the changes in the innermost layer of media are gradual, with an increase in thickness and the CFA progressively becoming random. The morphology and phenotype of SMCs also change, and the content of Anx increases. (B) In contrast, the other layers of media retain their original structure. (C) From the onset of plaque formation, the CFA inside is random, with SMCs lacking orientation, and there is a marked increase in the levels of macrophages, MMPs, and Anx. These phenomena collectively suggest that CFA is closely associated with the development of AS and may serve as a potential diagnostic biomarker.

In conclusion, changes in CFA are closely associated with inflammatory responses, phenotypic transitions of SMCs, the expression of osteogenesis-related genes and proteins, and the formation of Cal (Fig. 6). These pathological changes are observed exclusively in regions with randomized CFA, clearly distinguishing them from areas with oriented fibers. Abnormal CFA thus serves as a critical marker of lesion development in AS, enabling the identification of disease onset, localization of affected regions, monitoring of disease progression, and evaluation of plaque stability, which provides practical value for the early prediction and prevention of major cardiovascular events.

## Methods

### Feeding of ApoE^−/−^ mice

Seven-week-old male ApoE^−/−^ mice (Beijing Vital River Laboratory Animal Technology Co. Ltd.) were fed a high-fat diet (HD012a, Beijing Botai Hongda Biotechnology Co. Ltd.). The mice were housed 5 per cage with free access to food and water. Lighting was controlled according to natural sunrise and sunset cycles, and bedding (corncob, from Beijing Vital River Laboratory Animal Technology Co. Ltd.) was changed every 3 d. Every 5 weeks, 1 cage of mice was selected, and the mice were euthanized by cervical dislocation. The aortic arch was then collected and fixed in 4% paraformaldehyde (Wuhan Servicebio Biotechnology Co. Ltd.) for subsequent paraffin embedding.

This experiment strictly adhered to the provisions of animal welfare and rights protection laws. Since no surgical procedures or other interventions requiring anesthesia were performed during the course of the experiment, anesthetics were not used. Euthanasia of the mice was conducted via cervical dislocation, a method that complies with established regulations. Therefore, no anesthetic agents were employed throughout the entire study. This experiment has been reviewed and approved by the ethics committee of biology and medicine of Beihang University, approval number: BM20220023.

### Tissue paraffin embedding and sectioning

The excised vessels were fixed in 4% paraformaldehyde (Wuhan Servicebio Biotechnology Co. Ltd.) at 4 °C for over 4 h and then washed with phosphate-buffered saline (PBS) buffer (Wuhan Servicebio Biotechnology Co. Ltd., pH 7.4). Tissues were dehydrated in graded ethanol (Sinopharm Chemical Reagent Co. Ltd.), cleared with xylene (Sinopharm Chemical Reagent Co. Ltd.), and embedded in paraffin (Jiangsu Shitai Laboratory Equipment Co. Ltd.) at 57 °C. After overnight storage at 4 °C, paraffin blocks were sectioned at 5 μm using a paraffin microtome (Shanghai Leica Instruments Co. Ltd.). Sections were flattened in 40 °C water (Jinhua Kedi Instrument), mounted on adhesive slides (Wuhan Servicebio Biotechnology Co. Ltd.), baked at 60 °C (Tianjin Laibore Instrument Equipment), and stored at room temperature for further use.

### H&E staining

After deparaffinization, tissue sections were treated with high-definition staining pretreatment solution (Wuhan Servicebio Biotechnology Co. Ltd.), then stained with hematoxylin (Wuhan Servicebio Biotechnology Co. Ltd.), and differentiated using differentiation solution (Wuhan Servicebio Biotechnology Co. Ltd.). After bluing, sections were stained with Eosin Y (Wuhan Servicebio Biotechnology Co. Ltd.), dehydrated in absolute ethanol, cleared with xylene, and mounted with neutral balsam. Under a bright-field microscope (Nikon, Japan), nuclei appeared blue, cytoplasm pink to red, and calcium deposits light blue.

### Masson staining

After sectioning and deparaffinization, tissue sections were treated with mordant solution (Wuhan Servicebio Biotechnology Co. Ltd.) overnight at room temperature. Staining was performed sequentially with Celestine Blue, Mayer’s hematoxylin, acid differentiation solution, Ponceau Fuchsin, phosphomolybdic acid, and Aniline Blue (all from Wuhan Servicebio Biotechnology Co. Ltd.). Sections were then rinsed, treated with weak acid solution, dehydrated in graded ethanol, cleared with xylene, and mounted with neutral balsam (Sinopharm Chemical Reagent Co. Ltd.). Under a bright-field microscope, Col fibers appeared blue, while muscle fibers, cytoplasm, and red blood cells showed varying shades of red.

### Sirius Red staining and polarized light microscopy observation

Before staining, iron hematoxylin solution (Wuhan Servicebio Biotechnology Co. Ltd.) was prepared and applied to deparaffinized sections for staining, followed by rinsing. Sections were then stained with Sirius Red solution (Wuhan Servicebio Biotechnology Co. Ltd.), dehydrated in graded ethanol, cleared with xylene, and mounted with neutral balsam (Sinopharm Chemical Reagent Co. Ltd.). Under a bright-field microscope, Col fibers appeared red. Under polarized light, Col type I appeared bright orange-red, while type III appeared green.

### Alizarin Red staining

After deparaffinization and air-drying, the tissue sections were stained with Alizarin Red solution (Wuhan Servicebio Biotechnology Co. Ltd.) for 5 min. Following a rinse with distilled water, the sections were dehydrated, cleared, and mounted with neutral balsam. Under a bright-field microscope, calcium deposits appear as orange-red.

### Von Kossa staining

After deparaffinization and air-drying, sections were circled and stained with Von Kossa silver solution (Wuhan Servicebio Biotechnology Co. Ltd.) and then exposed to ultraviolet light for 4 h. After rinsing, sections were counterstained with hematoxylin, differentiated, blued, and stained with eosin. Dehydration was performed with graded ethanol, followed by xylene clearing and mounting with neutral balsam. Under a bright-field microscope, calcium salts appeared red-brown or black, nuclei blue, and the matrix pink to red.

### IHC

Sections were deparaffinized using an environmentally friendly solution (Wuhan Saiweier Bio-tech Co. Ltd.) and rehydrated through graded ethanol. Antigen retrieval was performed using citrate and EDTA buffers (Wuhan Saiweier Bio-tech Co. Ltd.). After cooling, slides were washed with PBS (Wuhan Saiweier Bio-tech Co. Ltd.) and then incubated with 3% hydrogen peroxide (Anjieke Science & Technology) to block endogenous peroxidase activity. Sections were blocked with 3% bovine serum albumin, followed by overnight incubation at 4 °C with appropriately diluted primary antibody (Abcam). After PBS washes, sections were incubated with secondary antibody and then developed with 3,3'-diaminobenzidine solution (Wuhan Saiweier Bio-tech Co. Ltd.) until brown staining appeared. Slides were counterstained with hematoxylin, differentiated, blued, and dehydrated through graded ethanol, butanol, and xylene. Finally, sections were mounted with mounting medium (Wuhan Saiweier Bio-tech Co. Ltd.). Under a bright-field microscope, nuclei appeared blue and positive signals brown.

### SEM

The section thickness was adjusted to 30 μm during sectioning. After standard processes of spreading, baking, and deparaffinizing to water, conductive adhesive was used to detach the tissue from the glass slide and attached to the sample stage. The sample was placed on the ion sputter coater (MC1000, Hitachi), and gold coating was performed for approximately 30 s. The sample is then ready for observation using a SEM (SU8100, Hitachi).

### Statistical analysis

Screenshots of stained section images were examined and captured using Case Viewer. Statistical analysis of the relevant images was performed with ImageJ. Quantitative data are expressed as mean ± SD, with *n* indicating the number of independent experiments. Data normality and between-group variance are assessed using the statistical package in GraphPad Prism. Statistical differences between groups are analyzed using Student’s *t* test, with significance levels set at *P < 0.05, **P < 0.01, and ***P < 0.001.

## Data Availability

The data that support the findings of this study are available within the article and the Supplementary Materials. All other relevant source data are available from the corresponding authors upon request.
