# Supplementary material for: Evolutionary Patterns of Collagen Fiber Arrangement and Calcification in Atherosclerosis
Source: Research (Wash D C). 2025 Jul 31;8:0798. doi: 10.34133/research.0798 (PMC12311368; doi:10.34133/research.0798)
Supplement: Supplementary 1 — Figs. S1 to S6 [file research.0798.f1.docx]

Supplementary Materials for

**Evolutionary Patterns of Collagen Fiber Arrangement and Calcification in Atherosclerosis**

**This PDF file includes:**

Figs. S1 to S6


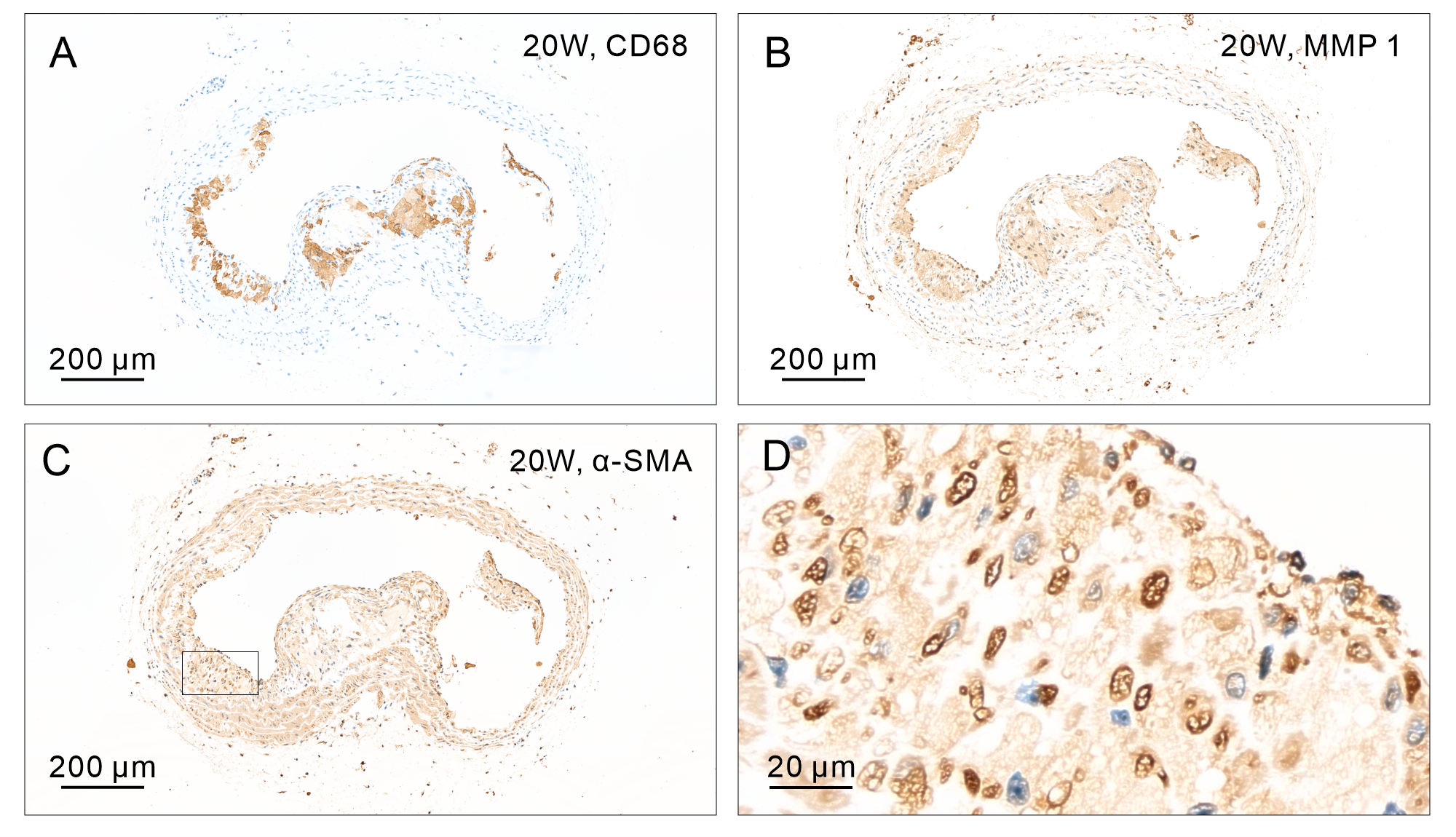


Fig. S1. Regions with high levels of macrophages and their secreted MMP1 are associated with the disorientation of SMCs. (A), IHC image of CD68. (B), IHC image of MMP1. (C), IHC image of α-SMA. (D), Magnified image of the region indicated by the black box in C.


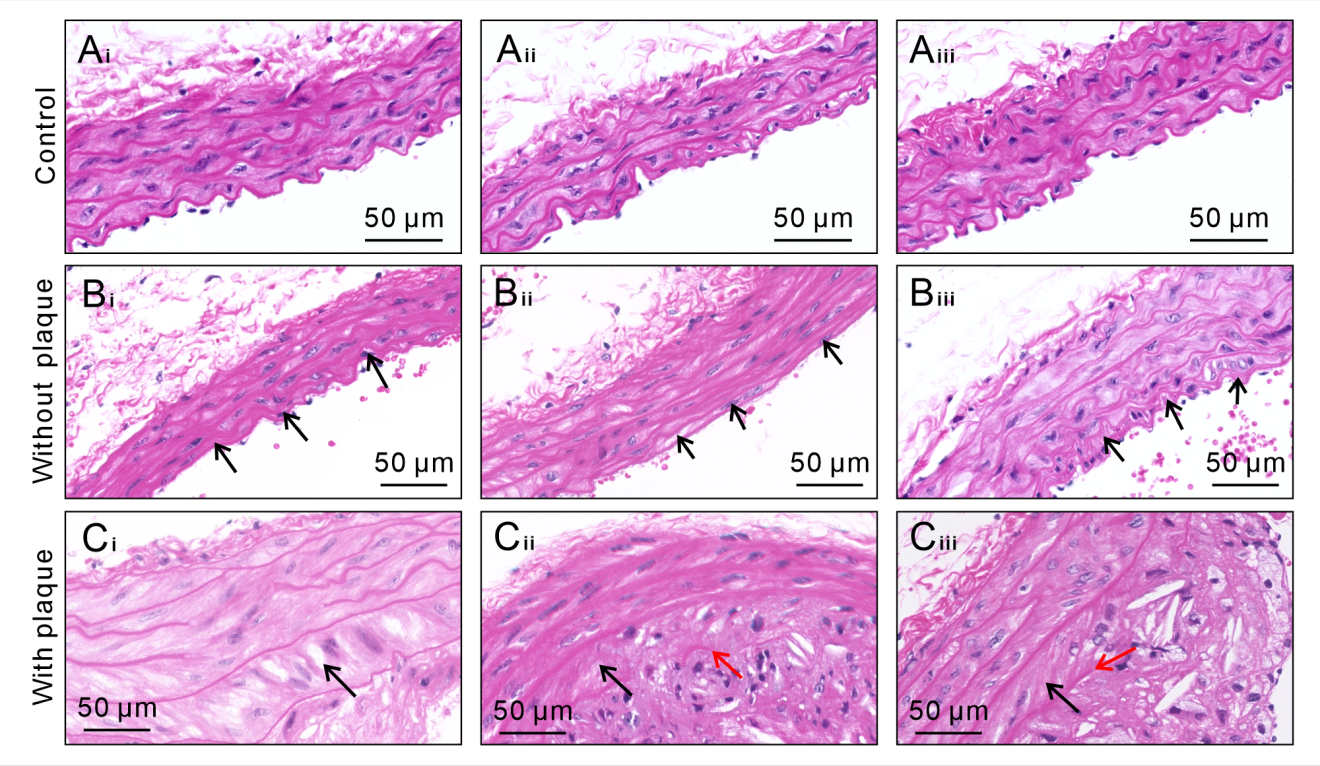


**Fig. S2. Changes in the innermost layer of the media.** (**A**), H&E staining images of the vascular wall in mice at 0 weeks on a high-fat diet. (**B**), H&E staining image of the vascular wall without plaque, showing no significant differences compared to the control group. (**C**), H&E staining image of the vascular wall with plaque, where the innermost layer of the media shows obvious lesions and severe damage.


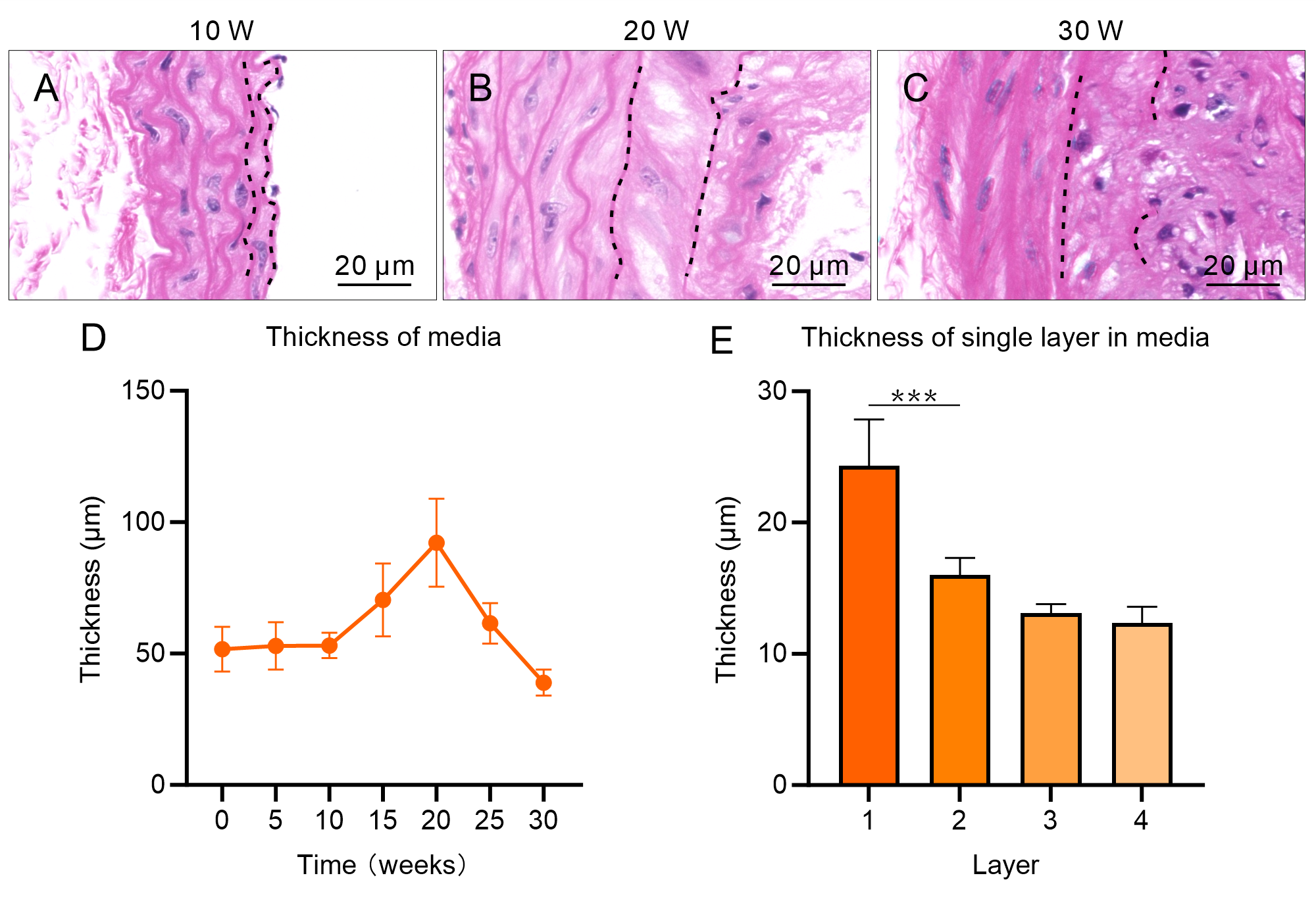


**Fig. S3. Changes in the media with disease progression.** (**A**), H&E staining of the media at week 10. (**B**), H&E staining of the media at week 20. (**C**), H&E staining of the media at week 30. (**D**), Statistical changes in the total thickness of the media over time. The increase in the overall thickness of the media is due to the thickening of its innermost layer, while the decrease in the media thickness is caused by the complete destruction of the innermost layer. (**E**), Statistical analysis of the thickness of each layer of the media at week 20. The innermost layer of the media, where the lesion occurs, is significantly thicker than the other layers (n=5).


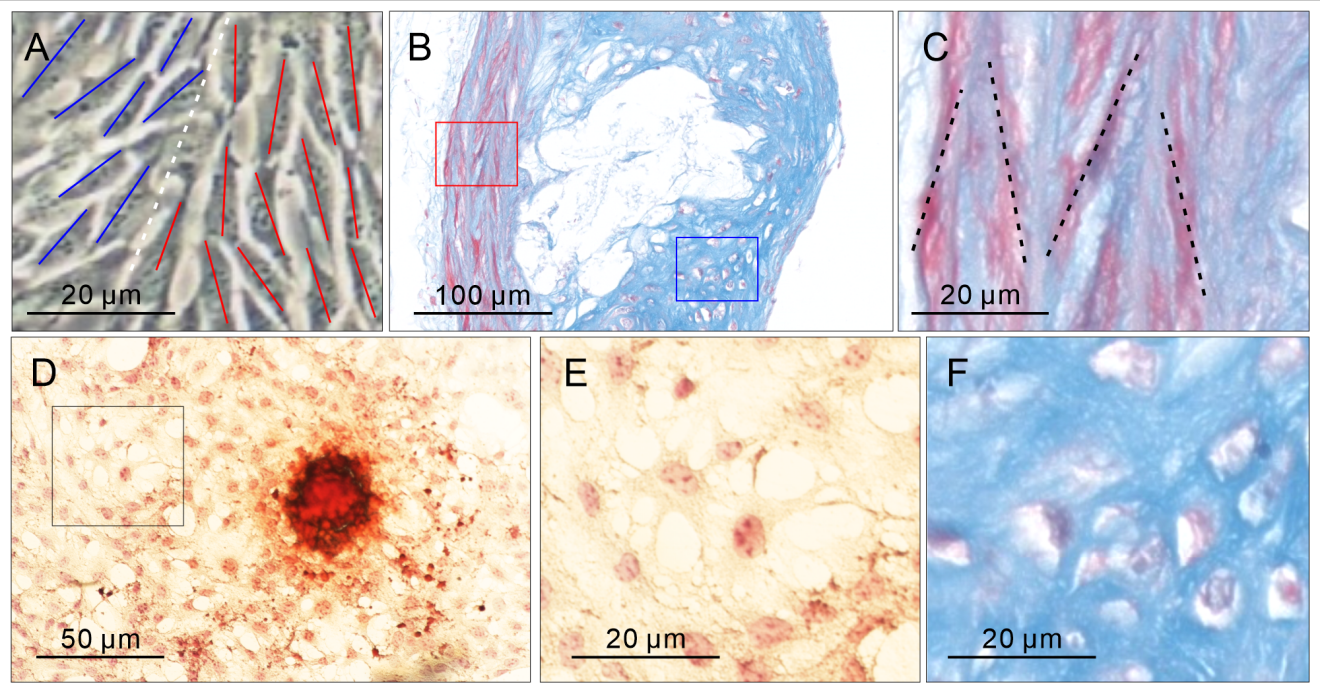


Fig. S4. Osteogenic differentiation of SMCs leads to a loss of orientation. (A), Morphology of SMCs without osteogenic induction, where the SMCs naturally form an orientation. (B), Masson staining image of the vascular wall at 30 weeks, with red box indicating the oriented region and blue box indicating the random region. (C), The morphology of muscle fibers in the oriented region of B is similar to that of SMCs shown in A. (D), Alizarin Red staining of SMCs 14 days after osteogenic induction, showing the formation of calcified nodules. (E), Magnified image of the black box region in D, showing that SMCs have lost orientation. (F), Magnified image of the random region in B, which closely resembles the structure shown in E.


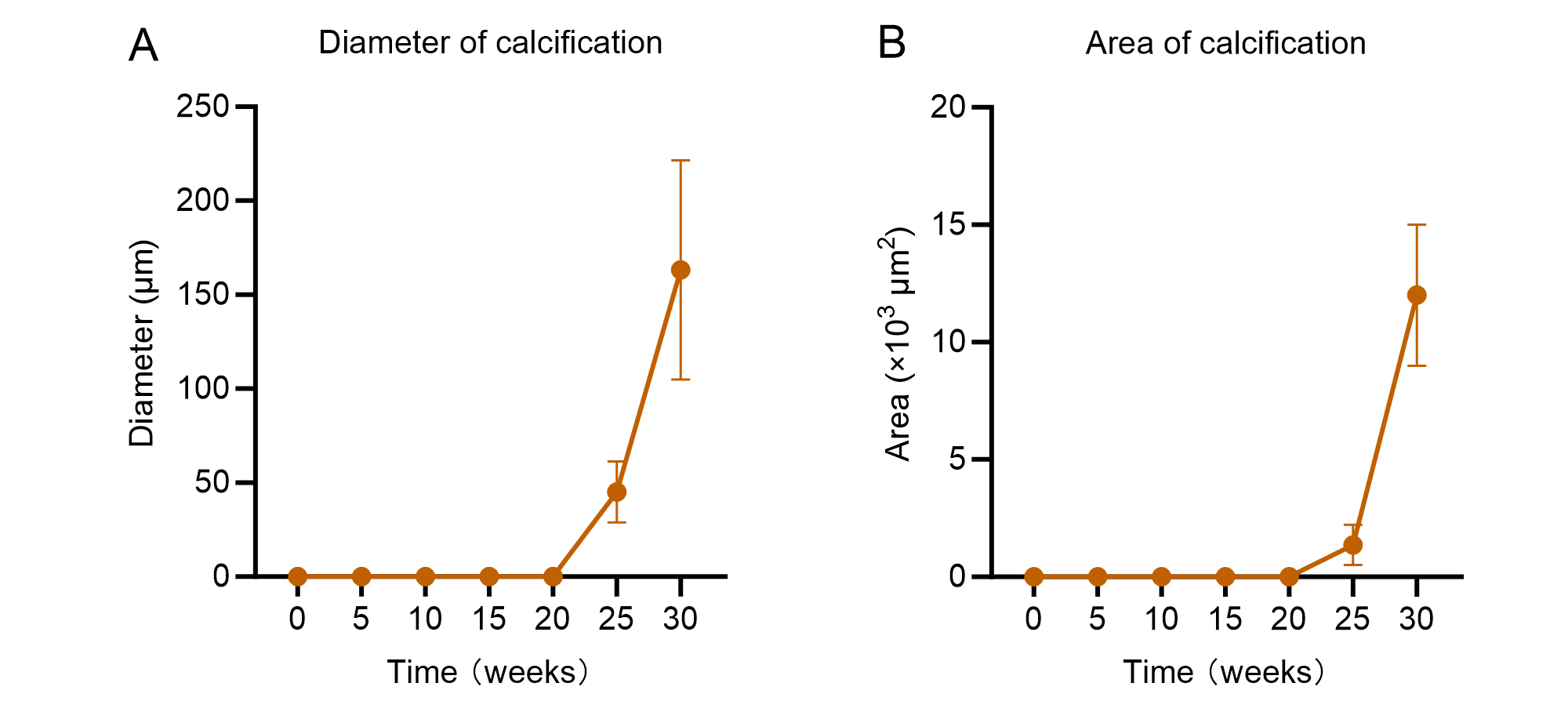


**Fig. S5. Statistical changes in the diameter and area of Cal over time.** (**A**), Statistical changes in the diameter of Cal over time (n=5). (**B**), Statistical changes in the area of Cal over time (n=5).


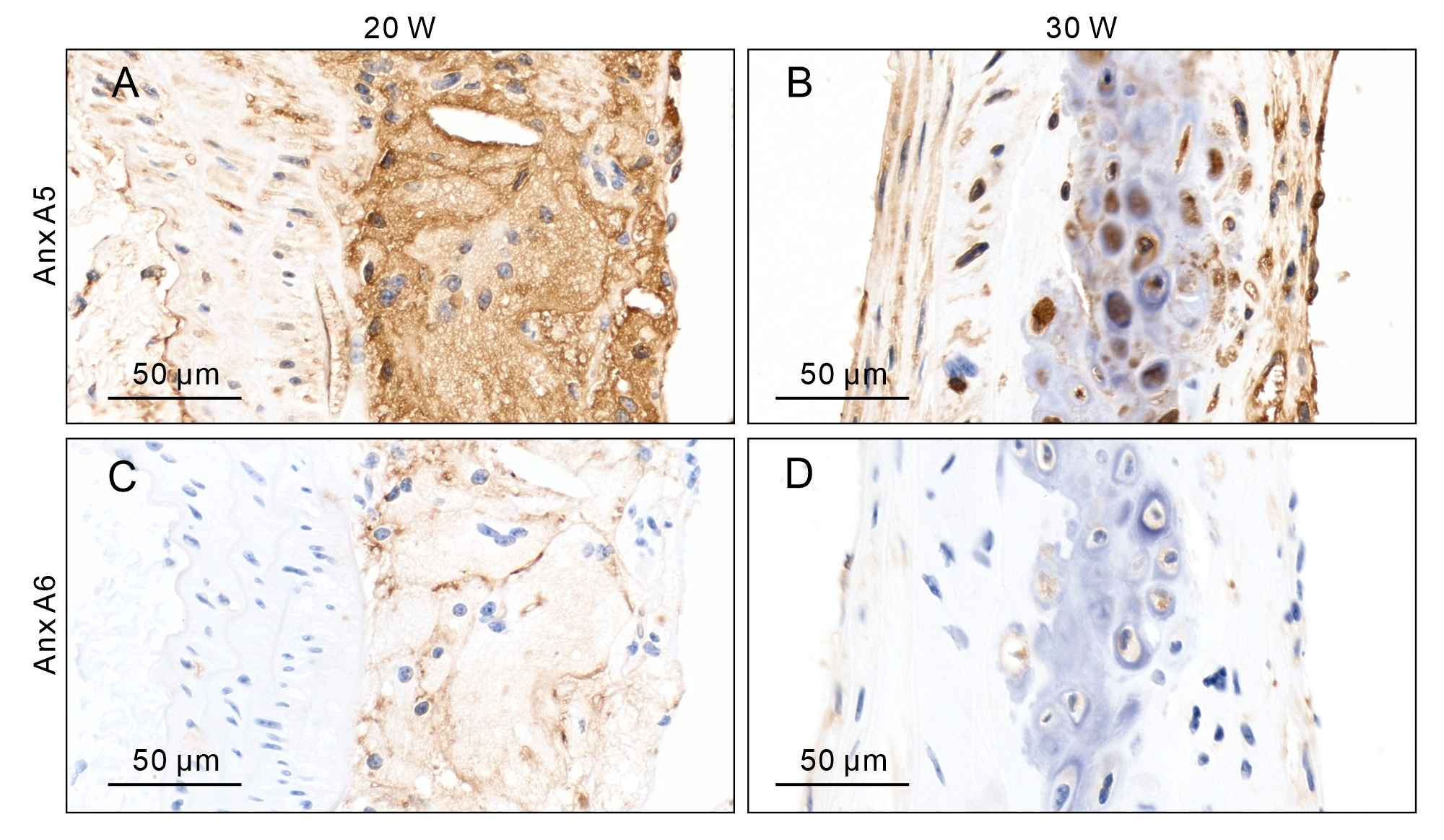


**Fig. S6. Anx is primarily observed before Cal formation.** (**A**), IHC of Anx A5 at week 20. (**B**), IHC of Anx A5 at week 30. (**C**), IHC of Anx A6 at week 20. (**D**), IHC of Anx A6 at week 30. After calcification occurred, the content of Anx was significantly reduced.
